# Centers for Disease Control and Prevention Yellow Book 2018: Health Information for International Travelers

**DOI:** 10.4269/ajtmh.17-0987

**Published:** 2018-04

**Authors:** Christopher A. Sanford

**Affiliations:** Departments of Family Medicine and Global Health; University of Washington; Seattle, Washington; E-mail: casanfo@uw.edu

Since its first publication in 1967 as a small pamphlet, the Centers for Disease Control and Prevention’s *Health Information for International Travel*, more commonly known as the Yellow Book, has blossomed into a 667-page, 7 × 10 inch paperback compendium of detailed, pragmatic, evidence-based recommendations on travel health. Although written first and foremost for clinicians (physicians, nurses, and pharmacists), much or most of the guide will be comprehensible to the lay traveler. Now published every other year ($49.95, University of Oxford Press), the Yellow Book is also available online, as an eBook, and as a mobile app.

The 2018 edition is largely similar to the 2016 edition. A few sections have been dropped (e.g., Perspectives on the Role of the Traveler in the Translocation of Disease, and Fear of Vaccines); others have been added (including Antibiotics in Travelers’ Diarrhea—Balancing the Risks and Benefits; and, in the chapter on Advising Travelers with Specific Needs, a section on Immunocompromised Travelers). Length, at 667 pages, is eight pages longer. The organization of chapters is unchanged.

The chapter on the pretravel consultation, in addition to listing the many topics that must be discussed before international travel, features a number of sections termed Perspectives, including discussions of travelers’ perceptions of risk, prioritizing care for the resource-limited traveler, cost analyses to justify the pretravel consultation, and the pros and cons of travelers self-treating travelers’ diarrhea with antibiotics. This chapter also contains helpful sections on altitude illness; jet lag; motion sickness; food and water precautions; protection against mosquitoes, ticks, and other arthropods; and other salient topics.

Chapter 3, by far the longest chapter in the book, details a number of infectious diseases, from amebiasis to Zika. The country-specific information regarding malaria prophylaxis and yellow fever vaccination is particularly helpful for practicing clinicians who need to make decisions about recommendations during a clinical encounter.

Although the country-specific information on malaria designates each malaria-endemic country’s estimated relative risk of malaria for U.S. travelers as being high, moderate, low, or very low, the relatively brief discussion of factors to take into account when deciding whether to advise the traveler to take a prophylactic medication, as opposed to insect precautions only (pp. 239–240 and p. 374), could be expanded.

A welcome addition in recent years has been the inclusion of 15 country-specific malaria maps and a number of yellow fever maps as well. This chapter also contains a number of historical overviews of selected topics, for example, “A History of Yellow Fever Vaccination Requirements,” “A History of Polio Eradication Efforts,” and “A History of Malaria Chemoprophylaxis,” which are interesting and informative for travel medicine buffs.

Chapter 4 discusses 16 common destinations for tourists and other travelers—this is a relatively recent addition to the Yellow Book—including East African safaris; Peru, including Cusco and Machu Picchu; and Saudi Arabia: Hajj/Umrah Pilgrimage (Cambodia, and Egypt and Nile River Cruses have been removed from the print edition but are available in the online version.) These well-informed sections consist of focused discussion of risks to health at specific destinations.

Chapter 5, Post-Travel Evaluation, discusses general considerations when seeing returned ill travelers and pragmatic discussion of workup of common complaints in returned travelers, including fever, prolonged travelers’ diarrhea, and skin and soft tissue infections.

Chapters 6, 7, and 8 address conveyance and transportation issues, travel with infants and children, and advising travelers with specific needs. Given the increasing numbers of travelers who are elderly and/or have specific medical needs, the topic of travelers with ongoing medical issues is particularly relevant to pretravel providers.

Although the sections on noninfectious threats have become longer in recent years, these sections are still relatively brief, given the importance of noninfectious threats to travelers. The chapter on injury prevention is less than five pages, which is less than 1% of the total length of this book. In defense of editors of the Yellow Book, research showing efficacy of interventions that reduce the risk of noninfectious threats to travelers lags behind research on reducing risk from infectious diseases. Nonetheless, given that the title of the book is *Health Information for International Travel*, not *Health Information on Infectious Threats to International Travelers—*and that road traffic injuries alone are the cause of as many as 25% of deaths of travelers—this section could benefit from expanded discussion of epidemiology, contributory factors, and strategies to mitigate risk of road traffic injuries, drowning, falls from height, and other noninfectious threats. Many of the infectious diseases discussed in Chapter 3 are extremely rare in travelers; possibly some of these sections could be shortened to allow expanded discussion of more common threats to travelers, including road traffic injuries. In addition, a section on lesbian, gay, bisexual, and transgender travelers would be a welcome addition.

The Yellow Book remains the best single source of information for clinicians who provide care to international travelers. Its tone is reasoned and reasonable; its implicit level of concern—cautious but not paranoid—is ideal. Increased attention to noninfectious threats to travelers would make it better still.

